# Bioinspired detoxification of blood: The efficient removal of anthrax toxin protective antigen using an extracorporeal macroporous adsorbent device

**DOI:** 10.1038/s41598-018-25678-0

**Published:** 2018-05-14

**Authors:** Ganesh Ingavle, Les Baillie, Nathan Davies, Nigel Beaton, Yishan Zheng, Sergey Mikhalovsky, Susan Sandeman

**Affiliations:** 1 School of Pharmacy and Biomolecular Sciences, University of Brighton, Brighton, BN2 4GJ United Kingdom; 2 School of Pharmacy and Pharmaceutical Sciences, Cardiff University, Cardiff, CF10 3NB United Kingdom; 3 Institute for Liver and Digestive Health, University College London, London, NW3 2PF United Kingdom

## Abstract

Whilst various remedial human monoclonal antibodies have been developed to treat the potentially life-threatening systemic complications associated with anthrax infection, an optimal and universally effective administration route has yet to be established. In the later stages of infection when antibody administration by injection is more likely to fail one possible route to improve outcome is via the use of an antibody-bound, adsorbent haemoperfusion device. We report here the development of an adsorbent macroporous polymer column containing immobilised *B*. *anthracis exotoxin-specific* antibodies, PANG (a non-glycosylated, version of a plant-produced human monoclonal antibody) and Valortim (a fully human monoclonal N-linked glycosylated antibody), for removal of anthrax protective antigen (PA) from freshly frozen human plasma and human whole blood. In addition, we have demonstrated that continuous extracorporeal blood recirculation through a Valortim-bound haemoperfusion column significantly reduced the blood plasma concentration of anthrax PA over 2 hours using an *in vivo* PA rat infusion model. This work provides proof-of-concept evidence to support the development of such alternative detoxification platforms.

## Introduction

Anthrax infection is caused by exposure to the micro-organism *Bacillus anthracis* (*B. anthracis*)^1^. The virulence of the bacteria is due primarily to the production of a tripartite toxin which as the name suggests is comprised of three elements: a non-toxic cell receptor binding protein known as “protective antigen” (PA) and two catalytic proteins known as “lethal factor” (LF) and “edema factor” (EF). The toxin follows the AB model, the A moiety comprising the catalytic subunits LF and EF, while the B moiety, PA, serves to translocate either EF or LF into the cytosol^2^. The B moiety is so named owing to its role as the key protective immunogen in the current human vaccine. It has been proposed that PA binds to ubiquitous cell surface receptors, at least two of which have been identified, anthrax/tumour endothelial marker 8 and capillary morphogenesis protein, whereupon it is cleaved by the cell-surface protease, furin, to expose the A moiety binding site^3,4^. Following proteolytic activation, PA forms a membrane-inserting heptamer that translates LF and EF into the cytosol. There is experimental evidence to suggest that this might not be the only model of toxin interaction and uptake^5^. In addition to forming protein complexes on cell surfaces, PA and LF are able to form a biologically active complex in the serum of a range of animal species, including primates, which is able to kill susceptible macrophages^5,6^.

Given the central role of this toxin in the disease process it is not surprising that the current recommended treatment for infected individuals is a prolonged regimen of antibiotic treatment combined with immunisation with a PA-based vaccine to eliminate any residual traces of the pathogen due to the ability of spores to persist in the lungs^7,8^. To be effective treatment must be started as soon as possible as animal studies have shown that once the level of circulating toxin exceeds a critical threshold antibiotics are no longer likely to be effective^9,10^. The current countermeasures for anthrax are vaccines, antibiotics and antibodies. They are an integral part of medical care, but all have limitations. Vaccines are an effective means of protection provided the individual has had the time necessary to generate a protective, antibody-based response^11^. Antibiotics have the advantage that they can be used to both protect and treat post-exposure by controlling the bacterial infection, but they fail to clear released toxins from the bloodstream, and antibiotic-resistant strains may not be effectively thwarted^12^.

Over the past decade, extensive research has been undertaken to develop therapeutic antibodies which target anthrax toxin components and can provide protection when given alone or in combination with antibiotic treatment^13–16^. Several recombinant monoclonal antibodies (mAbs) such as PANG^17^, Valortim^18,19^, and Raxibacumab^20^ have been developed which bind to PA and have been shown to protect infected animals. They act by inhibiting different stages of the intoxication process, including the inhibition of PA-receptor binding, proteolytic cleavage, oligomerisation, internalisation, and EF/LF binding. Raxibacumab which has been approved by the FDA for the treatment of inhalational anthrax using the Animal Rule (US Food and Drug Administration 2012) increases survival in animal models when given pre-exposure^21^ and has demonstrated a survival benefit for exposed rabbits when administered in combination with antibiotics^22^.

Given the paucity of data on human inhalation of anthrax the post-exposure period for effective treatment of human disease remains unknown. In an effort to address this issue, Rubinson *et al*. extrapolated animal model data and estimated that that the co-administration of raxibacumab and antibiotic within 7 days of spore exposure would protect the majority (>80%) of individual^23^. To derive this estimate the authors made assumptions about the health status of the individuals prior to infection and while these assumptions may be appropriate for segments of society such as the military, they are unlikely to hold true for the vast majority of the civilian population. Indeed, while treatment with the antibody might completely neutralise the toxin, this alone may be insufficient to ensure the survival of infected individuals. Studies undertaken in animals have shown that infection with *B. anthracis* elicits many features of a septic disease and that while the toxin plays a central role in pathology the host’s own uncompensated inflammatory and coagulopathic responses also contribute to the process^24,25^. For example the release of *B.anthracis* cell wall peptidoglycan has been shown to trigger the production and release of pro-inflammatory TNF-alpha and IL-8^26^. We hypothesize that a system capable of removing both PA and pro-inflammatory mediators would enhance the chance of survival of infected individuals. To advance the development of such a system we are exploring the feasibility of attaching PA specific monoclonal antibodies to the surface of the cyrogel in a manner which optimizes its ability to remove PA from circulating blood.

Chemical immobilisation of monoclonal antibodies onto a solid supporting surface can be carried out using a number of approaches for selective purification of biological media^27^. Factors such as the size, surface chemistry, surface density and orientation of the immobilised antibodies strongly affects immunosorbent efficacy^28–35^. Immobilization should be done in a way that does not block the antibody’s active sites during antibody-antigen interaction. Protein-A is commonly used in antibody immobilization to correctly orientate the antibody through bond formation with the heavy chain region (Fc) of the antibody^33,34^. This leaves the flexible heavy and light chain regions (Fab) accessible for binding to antigen epitopes.

Blood detoxification by adsorption of anthrax toxins using chemically immobilised PA antibody bound to the internal surface of a macroporous interconnected cryogel column has yet to be explored. In our previous work, we investigated the synthesis of a range of macroporous cryogel adsorbents with tunable physical and mechanical properties for covalent immobilisation of anthrax toxin specific antibodies, PANG and Valortim, using protein-A affinity ligand. We demonstrated that protein-A bound poly(acrylamide-co-allyl glycidyl ether) (AAm-AGE) cryogels had the physical properties and capacity to bind antibody for the removal of anthrax toxin PA from phosphate buffered saline (PBS)^36^. In this article, we report for the first time the significantly increased removal of anthrax PA by covalently immobilised Valortim antibody on macroporous polyacrylamide-based cryogel columns from fresh frozen human plasma and freshly drawn human whole blood. In addition, a miniaturized extracorporeal circuit incorporating a plain and Valortim antibody bound cryogel, was used to filter blood in a Sprague-Dawley rat intravenous anthrax PA toxin infusion model in order to assess PA removal from blood over time.

## Results

### Material synthesis and porous morphology

**Figure 1:**
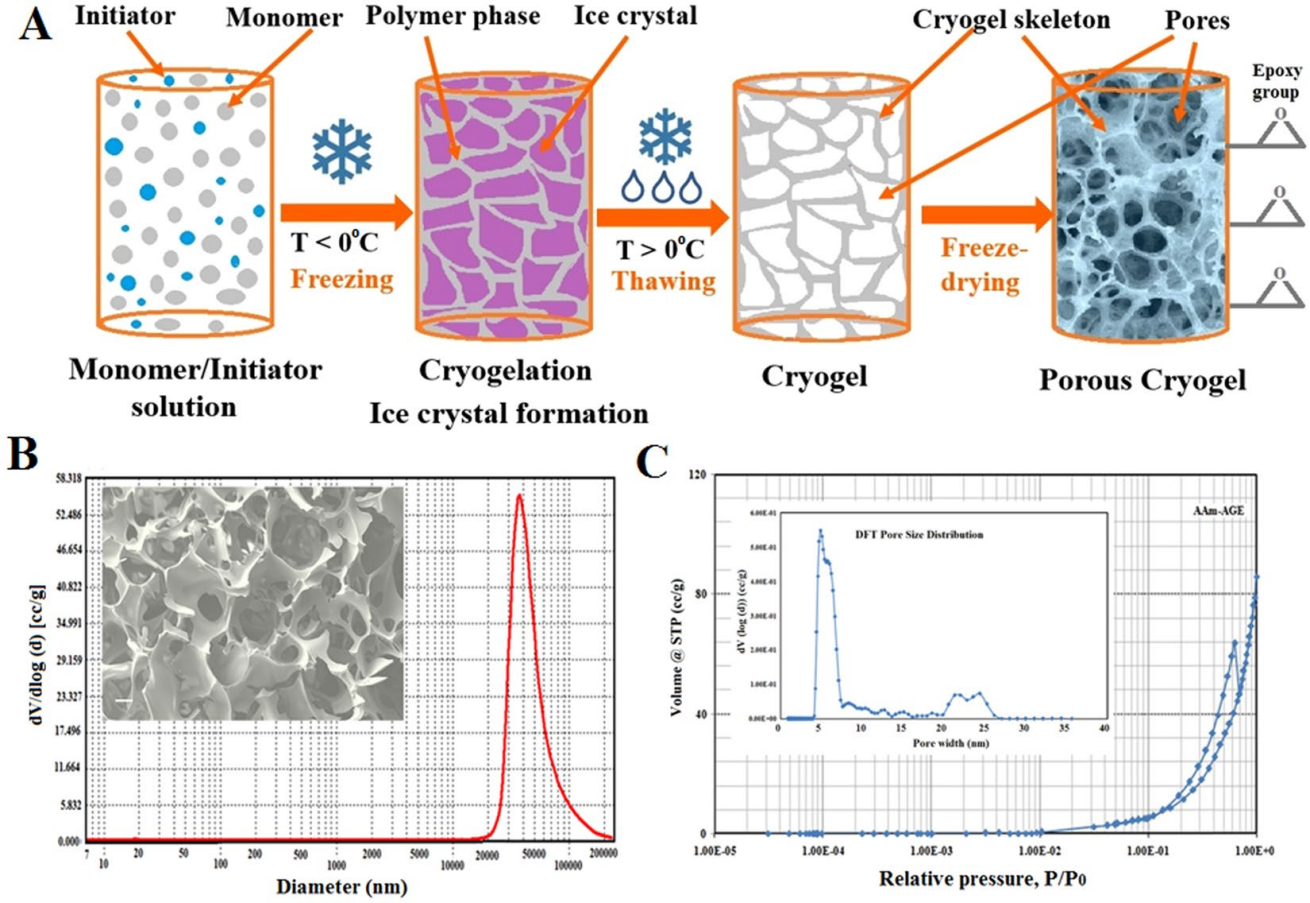
The process of cryogel formation and macro-meso pore size distribution. (**A**) The different stages that occur during cryogelation. (**B**) Macropore size distribution as determined by mercury porosimetry and inset SEM images showing internal porous architecture, Scale bar = 20 *μ*m. (**C**) N_2_ adsorption isotherm with inset image representing mesopore size distribution.

**Figure 2:**
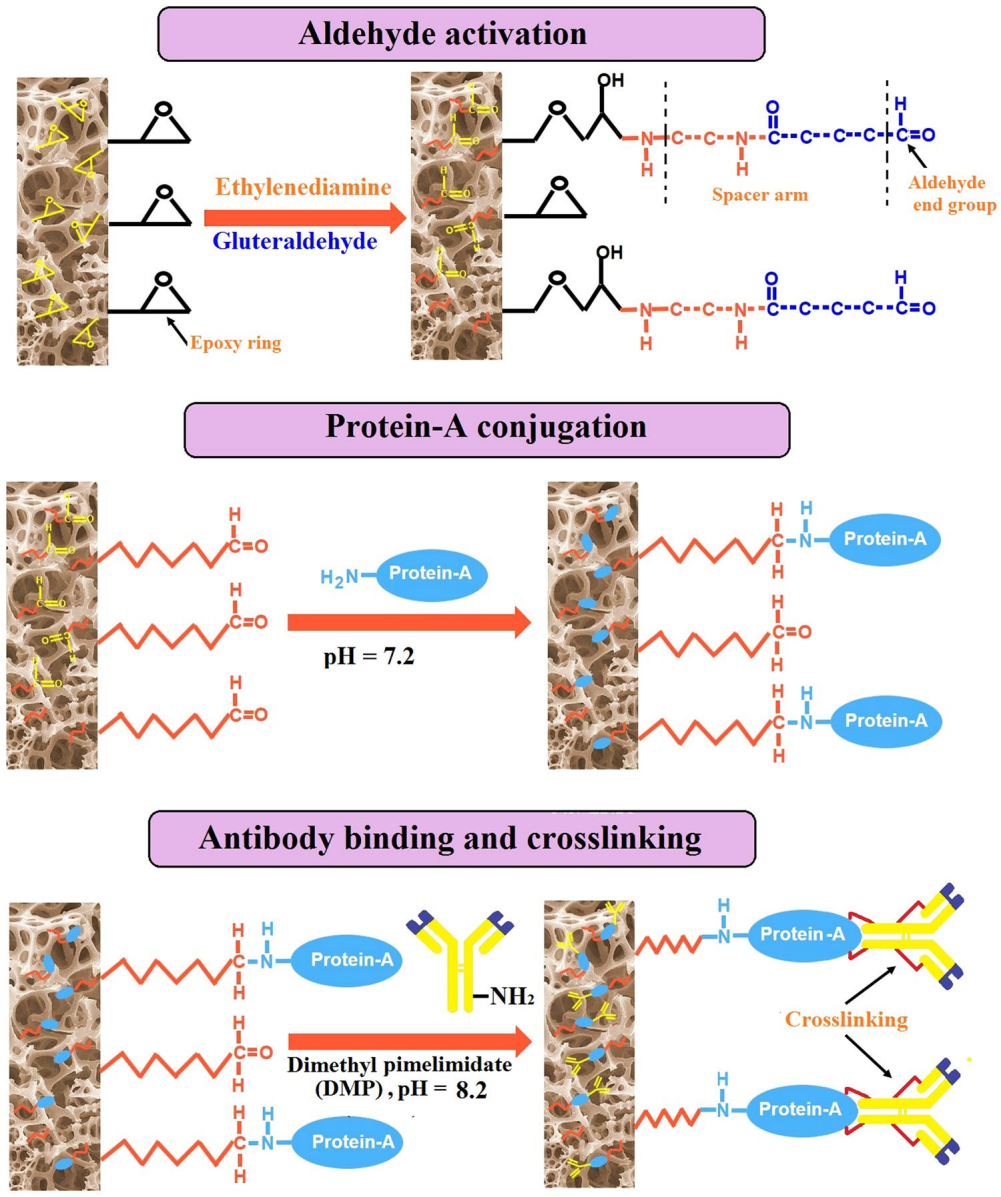
Schematic representation illustrating the glutaraldehyde activation approach utilized for protein-A conjugation and covalent immobilisation of monoclonal antibodies via protein-A on epoxy containing cryogel and subsequent crosslinking using dimethyl pimelimidate (DMP). Ethylenediamine was used as a donor of a spacer arm to improve binding between affinity ligand (protein-A) and antibody by overcoming any effect of steric hindrance.

Cylindrical macroporous poly(AAm-AGE) cryogel columns were synthesized by co-polymerisation of monomers, acrylamide (AAm) and allyl glycidyl ether (AGE), using a cryogelation technique (Fig. 1A)^36^. During co-polymerisation N, N’-methylenebis(acrylamide) (MBA) was used as a crosslinker, while N, N, N’, N’-tetramethylethylenediamine (TEMED) and ammonium persulfate (APS) were used as a redox initiator pair to activate polymerisation. Mercury porosimetry and scanning electron microscopy (SEM) indicated a consistent porosity in the 10–100 *μ*m range with smooth pore walls (Fig. 1B). Nitrogen adsorption isotherm measurements, obtained to characterise the porous structures of AAm-AGE cryogel samples, exhibited a typical type-IV sorption isotherm profile that are indicative of mesoporosity (Fig. 1C). The pore size distribution profile for AAm-AGE cryogel revealed that the cryogel predominantly possesses macropores (Fig. 1B) and with some evidence of meso-pores (Fig. 1C), which accounts for the high surface area of this material. The surface epoxy groups on the AAm-AGE cryogel were activated by glutaraldehyde which introduced aldehyde groups for covalent attachment of affinity ligand protein-A which was further used to link PANG and Valortim antibodies (Fig. 2)^36^.

### Cryogel binding capacity and the impact of glycosylation on PANG vs Valortim binding and PA capture

**Figure 3:**
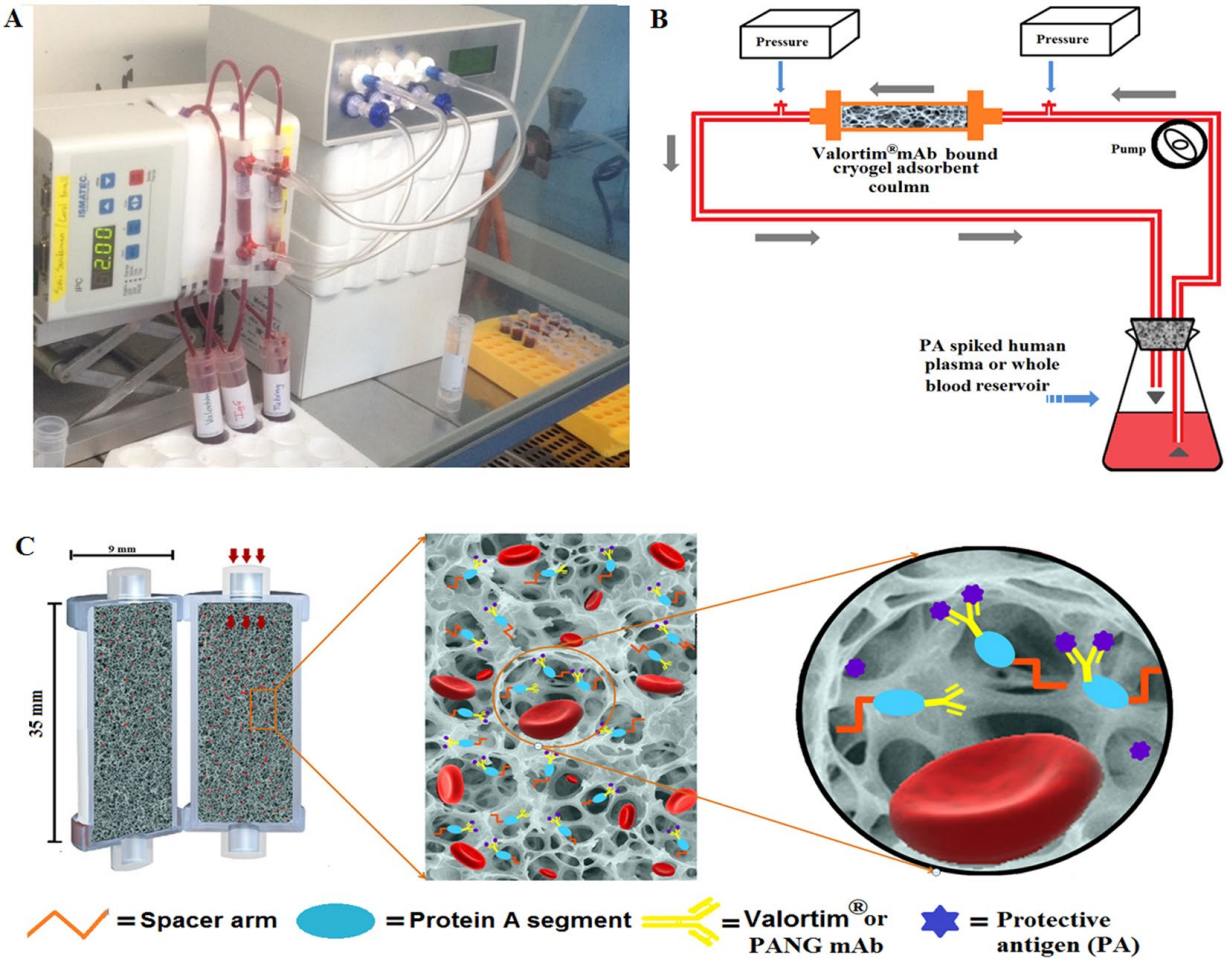
(**A**) Lab scale *in vitro* experimental setup for the circulation of PA-spiked whole blood (from healthy donors). (**B**) A schematic representation of the *in vitro* circuit utilized for blood perfusion studies. (**C**) Vertical cross section of cryogel adsorbent column sitting in a plastic casing and cartoon illustration displaying antibody-antigen interactions inside interconnected porous cryogel structure. The adsorbent device is disposable, consisting of antibodies covalently coupled to the cryogel surface through protein-A which is located throughout the interconnected pores. Anthrax protective antigen in the blood is transported via convection and diffusion through the 10–100 *μ*m pores in the cryogel matrix where they are bound.

**Figure 4:**
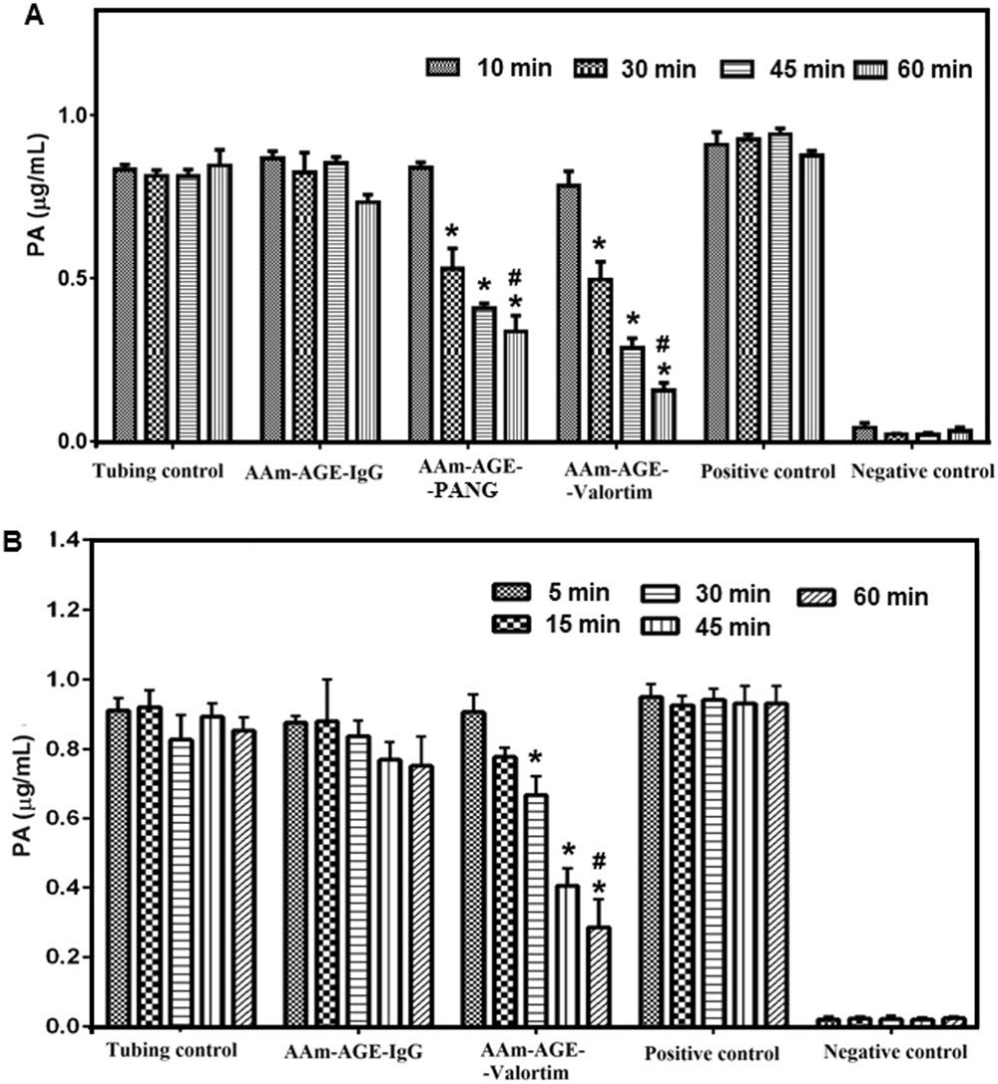
(**A**) Plasma and (**B**) whole blood PA concentration at indicated time points as determined by sandwich ELISA. Bar graphs shown are mean ± standard deviation (n = 3). Significant differences between cryogel groups were determined using a two-way ANOVA followed by Bonferroni’s post-hoc test to compare concentration of PA removed by cryogels. ‘*’Indicates significant different values within the same group (p < 0.05), while ‘#’values indicate statistically significant differences between the cryogel groups (p < 0.05).

The AAm-AGE cryogel has a large specific surface area (101 m^2^/g) on which proteins covalently attach with a high protein binding capacity (96.4 ± 10.4 mg/g of adsorbent)^36^. Previous work demonstrated that the glycosylated Valortim antibody (117 mg/g) bound significantly higher levels of PA from PBS (p < 0.05) than non-glycosylated PANG (108 mg/g) on the AAm-AGE-protein-A cryogel column^36^. *In vitro* PA removal studies from human plasma and blood were carried out using a lab scale continuous circulation model (Fig. 3A and B) connected to Valortim or PANG-bound cryogel columns capable of capturing anthrax toxin PA by antibody-antigen interaction (Fig. 3C). An anti-PA sandwich enzyme-linked immunosorbent assay (ELISA) was used to demonstrate that tubing and cryogel controls without PA bound antibody did not remove significant levels of PA from plasma at any time point over the 60-minute recirculation period (Fig. 4A). All four control groups showed similar PA values at all sampling time points. In contrast, over the 60-minute recirculation period a significant reduction in plasma PA concentration was observed for both the AAm-AGE-Valortim cryogel columns (1 to 0.15 *μ*g/mL) and the AAm-AGE-PANG cryogel columns (1 to 0.33 *μ*g/mL), with 85% and 67% reduction respectively. The final plasma PA concentration of the AAm-AGE-protein-A-Valortim group (0.15 *μ*g/mL) was significantly reduced when compared to both the non-specific IgG mAb-bound AAm-AGE column (0.73 *μ*g/mL) and the tubing control (0.84 *μ*g/mL). A similar significant reductions in final plasma PA concentration were observed for the AAm-AGE-protein-A-PANG group where a final concentration of 0.33 *μ*g/mL was obtained. The removal of PA from human whole blood by Valortim-bound AAm-AGE cryogels showed results that were similar to those seen in plasma (Fig. 4B). Both the tubing control and non-specific IgG mAb-bound AAm-AGE cryogel groups did not remove PA from blood over the 60-minute recirculation at any time point and both groups had similar PA values throughout. As for the plasma experiments, the AAm-AGE-Valortim cryogel columns removed a significant amount of spiked PA from whole blood, removing 60% (1 to 0.40 *μ*g/mL) and 72% (1 to 0.28 *μ*g/mL), at the 45 and 60-minute time points, respectively. This decrease in PA concentration was not significant at earlier time points, but does show an increased removal over time, an observation that is typical of a kinetic process such as this. When compared with the final PA concentration in the non-specific IgG mAb-bound AAm-AGE and the tubing control, the AAm-AGE-protein-A-Valortim cryogels significantly reduced the whole blood PA concentration by 62% (0.75 *μ*g/mL vs 0.28 *μ*g/mL, p < 0.05) and 67% (0.85 *μ*g/mL vs 0.28 *μ*g/mL, p < 0.05), respectively.

### *In vivo* studies to determine PA removal from rat blood

**Figure 5:**
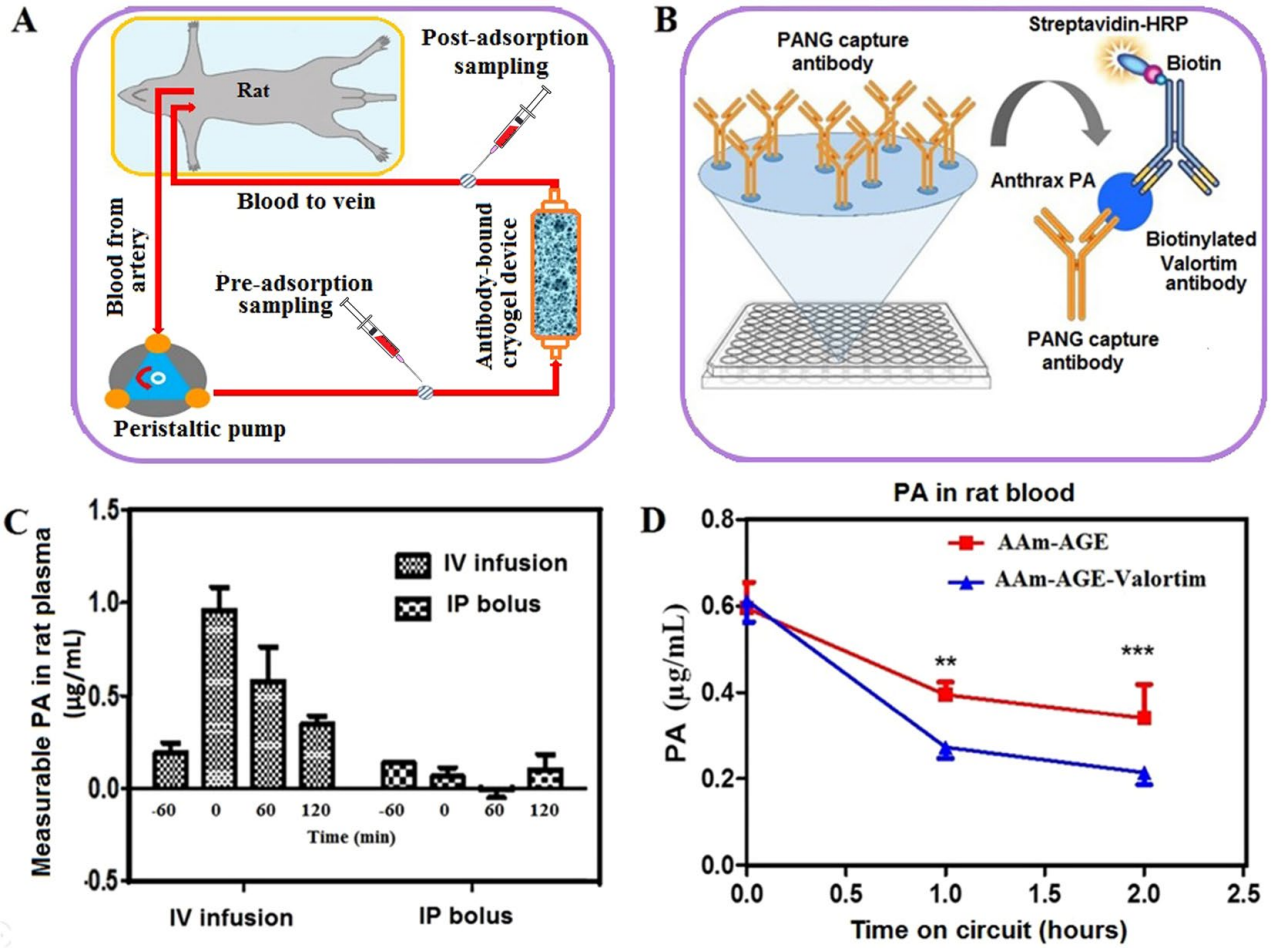
(**A**) An *ex vivo* extracorporeal circuit incorporating cryogel columns. (**B**) The principle of the anti-PA sandwich ELISA used to measure plasma PA concentrations of PA left in a rat blood. (**C**) Measurable rat plasma PA concentrations immediately after administration by IV infusion (time = −60 minutes) or IP bolus. Time 0 was set at 60 minutes post IP bolus or at the end of the infusion period, with the remaining samples taken at hourly intervals. (**D**) Plasma PA concentration in circulating rat blood at 0 (at the end of a 60-minute IV infusion; administered initially at 100 *μ*g/kg, followed by a maintenance infusion at 40 *μ*g/kg for the final 2 hours), 1 and 2-hour time points. Graphs shown are mean ± standard deviation (n = 6, p < 0.01).

**Figure 6:**
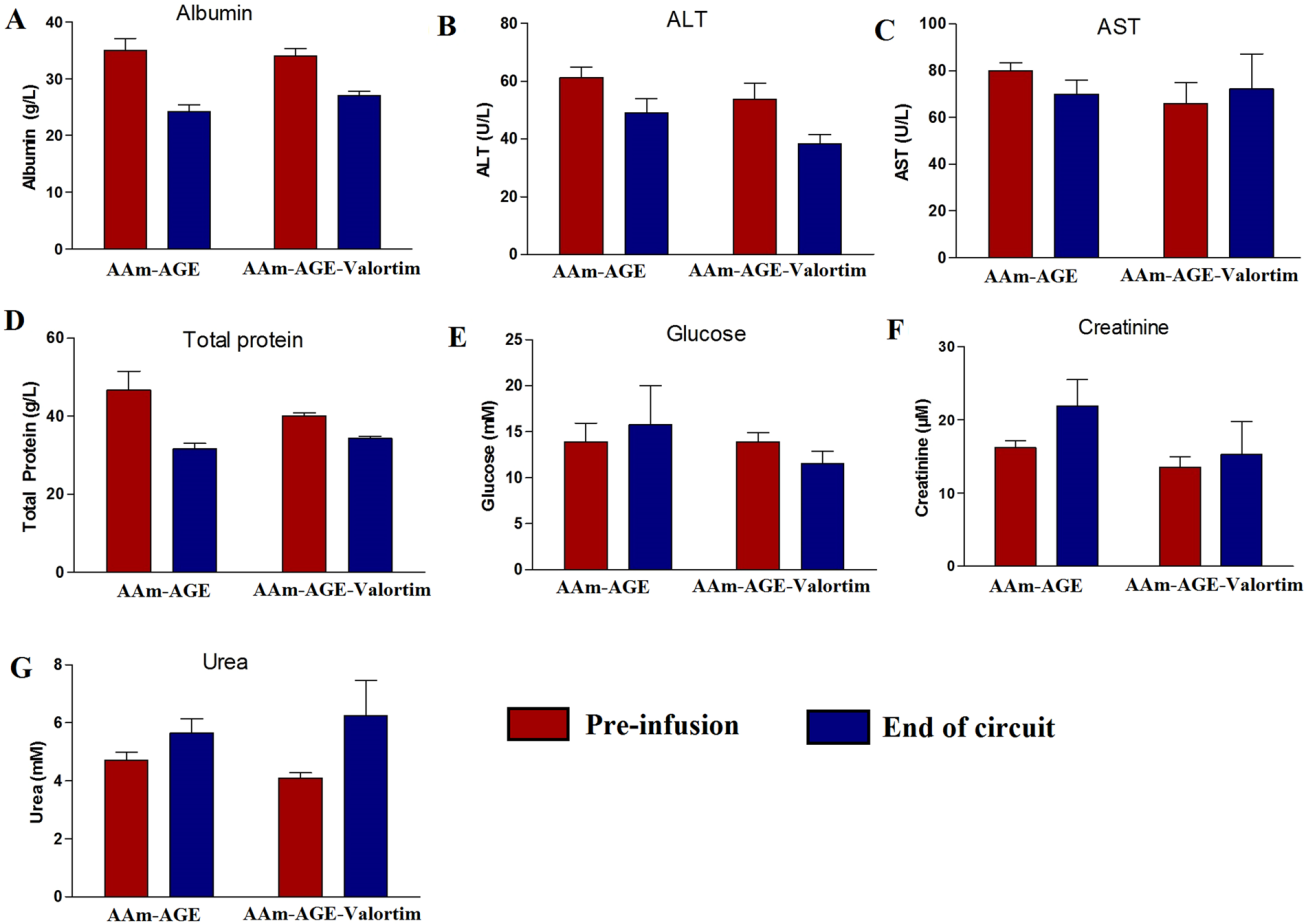
Biochemical analysis results of (**A**) albumin, (**B**) ALT, (**C**) AST, (**D**) total protein, (**E**) glucose, (**F**) creatinine, and (**G**) urea concentrations in rat blood measured before PA IV infusion and at the end of the circuit. No statistically significant differences were noted.

This work demonstrates that engineered antibodies crosslinked to cryogels bind PA toxin *in vitro*, and may be used as an effective means for *in vivo* PA toxin removal. To test this, miniaturized extracorporeal circuits incorporating either plain (AAm-AGE) or Valortim antibody-bound (AAm-AGE-Valortim) cryogel columns were set up in an *in vivo* rat model (Fig. 5A). Sprague-Dawley rats were infused intravenously with anthrax toxin PA and placed on extracorporeal circulation (2 hrs at 2 mL/min). Blood samples were collected at 0, 60 and 120 minutes and concentration of PA was measured by an anti-PA sandwich ELISA (Fig. 5B). It was first determined that intravenous (IV) infusion of PA toxin (100 *μ*/kg/hr over 1 hour) resulted in a significantly higher circulating concentration of PA in rats than intraperitoneal (IP) injection, although the circulating PA concentration decreased over the ensuing 2 hours, most likely due to tissue deposition (Fig. 5C). As a result, it was determined that a maintenance infusion of PA (40 *μ*g/kg/hr) was required to maintain elevated circulating PA toxin levels throughout the period of extracorporeal circuit treatment. Following the initial intravenous PA protein infusion (1 hr), the Valortim-bound column significantly reduced circulating PA concentrations by 79% (n = 5) over a subsequent 2 hr evaluation period, measured by an anti-PA sandwich ELISA (Fig. 5D). Over a 2-hour extracorporeal circuit treatment, a significant reduction in PA (p < 0.01) was observed in comparison with plain AAm-AGE columns (66%, n = 6) over the same time period (Fig. 5D). Further, various blood biochemistry analyte levels were monitored at the start and end of the circuit and showed no significant effects on blood biochemistry during the circuit treatment period (Fig. 6A–G). This data demonstrate that Valortim-bound cryogel columns are able to efficiently remove PA toxin from whole blood not only within *in vitro* studies, but also when used *in vivo* as an extracorporeal therapy.

## Discussion

The device, designed in the current study to be used extracorporeally like dialysis, consists of a Valortim antibody-bound, highly interconnected macroporous polymeric AAm-AGE cryogel. It traps circulating anthrax toxin component PA and thus reduces the impact of the potent tripartite toxin on the unfortunate host. The interconnecting macroporous polymer structure offers a large surface area for antibody attachment and is formed by cryo-polymerisation. During synthesis polymerisation occurs at sub-zero temperatures as a result of a complex process combining the crystallization of solvent (water) and the free radical co-polymerisation of AAm (monomer), MBA (crosslinker) and AGE. AGE is used as a minor co-monomer to introduce epoxy functional rings onto the porous cryogel surface and allow covalent attachment of the affinity protein-A^36^. Protein-A affinity ligand is linked to the epoxy-containing macroporous cryogel matrix with a 7-carbon spacer arm generated on the epoxy-activated porous surface using an ethylenediamine (EDA) and glutaraldehyde reaction, which is further used to attach PANG and Valortim antibodies using dimethyl pimelimidate (DMP). The size and structure of the cryogel pores are controlled by two processes that occur simultaneously. The first is crystallization during cryogelation which is controlled by freezing conditions and the second is polymerisation, which is influenced mainly by the concentration of monomers, cross-linkers and the initiator system^37–39^. Part of the monomer solution reserved in the voids around the ice crystals remains unfrozen due to the concentration of the dissolved substances. The co-polymerisation and the formation of the cryogel matrix occurs in the unfrozen phase. Finally, melting of entrapped ice create the system of interconnected pores (Fig. 1A). In order to be used as a matrix to capture anthrax toxin PA, cryogels should have large and interconnected pores of a size greater than 10–15 *μ*m. The study of pores in wet hydrogels presents a significant challenge^40^. Whereas the porosity and pore size distribution of a rigid porous material in the dry state are traditionally determined by mercury intrusion porosimetry^37,41^, there is no general method to determine pore size and pore structure in soft and highly hydrated systems.

We have demonstrated a synthesis route for an AAm-AGE cryogel which produces large pores, an interconnected channel structure and a large surface area allowing antibodies to bind for blood-borne toxin removal. The concept was first developed in our previous work optimizing the structural performance of the cryogels themselves. This ultimately resulted in a high protein-A binding capacity, enabling optimal antibody loading through control of excessive steric hindrance^36^. Glyco-engineered antibodies exploit the common post-translational modification of glycosylation in the antibody’s Fc region to modulate effector function and stability, and ultimately therapeutic efficacy. Antibody glycosylation, particularly in the Fc region of IgGs, has been extensively studied in health and disease and it has been shown that N-glycans are critical for antibody function. After 60 minutes recirculation, the glycosylated Valortim antibody bound significantly higher levels of PA (p < 0.05) than non-glycosylated PANG on the AAm-AGE-protein-A cryogel column using fresh frozen human plasma and freshly withdrawn blood suggesting a potential role for in explaining the disparity. Additionally, the Valortim-bound columns showed a significantly increased ability (p < 0.05) to clear PA from plasma with respect to PANG columns in comparison with the non-specific IgG mAb-bound AAm-AGE and tubing control, respectively confirming specific binding capacity. These results are in line with our initial finding of an increased protein binding capacity in Valortim versus PANG antibodies from PBS and strongly indicate that Valortim-bound cryogel columns are the more effective approach. Given this, only the Valortim-bound cryogel columns were used for the *ex vivo* studies. The efficiency of PA binding by cryogel immobilised antibodies is a combination of cryogel binding efficiency, correct orientation of the antibody with regards to protein-A interaction and the binding affinity of the antibody for its target on PA. From these studies, we can conclude that we are able to attach both glycosylated and non-glycosylated antibodies to a cryogel in a manner which allows them to capture their biological target offering options for further functionalisation. Results also suggest a potential role for glycosylation in device efficacy although this has yet to be confirmed beyond the correlation observed in this study.

To summarise, a novel *ex vivo* technique for antibody-based PA capture using a macroporous polymer cryogel column has been demonstrated. This approach presents new possibilities for the development of an effective antibody-based therapy against anthrax. We have shown using *in vitro* and *in vivo* models that a Valortim bound adsorbent haemoperfusion column can bind significant levels of PA from blood. While these results are promising further work is required to determine whether this approach is capable of effectively treating an infected individual. For example, by immobilising the antibody on the surface of the cryogel it is unlikely that it will be able to interact with PA which has already bound to host cells as is thought to happen when Valortim is delivered by injection^18^. The logical next step would be to compare the efficacy of this approach to that of the injected (unbound) Valortim with regards to its ability to protect against a lethal toxin challenge. Further studies will be needed to ascertain the survival benefit of the column in a combined PA + LF model, though this will require further ethical consideration as in order to achieve reproducible detection levels of the proteins, the doses required have previously been shown to be universally lethal.

Anthrax specific glyco-engineered antibody-bound cryogel columns offer post-exposure protection against *B. anthracis* infection, making them an attractive candidate for therapeutic preparations aimed at providing efficient and immediate anthrax toxin neutralisation. Glycosylated and immobilized PA capturing antibodies on a porous support may be of therapeutic value for alleviating the symptoms of anthrax toxin in infected individuals and for medium-term prophylaxis against infection. The ability to create cryogels which specifically bind individual molecules through the incorporation of targeted specific antibodies raises the possibility of developing multifunctional cryogels capable of capturing a range of sepsis related factors. In addition, other adsorbents may be incorporated within a composite cryogel to extend the target range to sepsis related key pro-inflammatory mediators^42^ since the removal of these and pathogen produced virulence factors during late stage infection has been shown to impact patient survival^43^.

The Valortim-bound cryogel prototype tested in this proof-of-concept study using an *in vivo* haemoperfusion model demonstrated significant depletion of PA levels from infected blood. Following further evaluation and technical development the device holds potential clinical interest particularly with the need for pertinent therapeutic agents against any conceivable future bioterrorism threats. Further, this approach to toxin capture via extracorporeal haemoperfusion may also provide an effective strategy for treating viral diseases caused by Ebola^44^, Zika, enveloped viruses including (therapy resistant) HIV, hepatitis B or C (induced liver failure), and even influenza.

## Methods

### Preparation of AAm-AGE cryogel and protein-A attachment

Monolithic macroporous poly(AAm-co-AGE) cryogels containing epoxy surface functionality were synthesised by cryogelation technique at −12 °C by mixing the monomers 0.954 g acrylamide (AAm, 99%, Sigma, St Louis, MO, USA), 0.266 g of N, N’-methylenebis(acrylamide) (MBA, 99%, Sigma, St Louis, MO, USA) and 0.358 mL of allyl glycidyl ether (AGE, 99% Sigma, St Louis, MO, USA) in deionized water at 8% w/v initial monomer concentrations. The monomer reaction mixture was transferred in to glass tubes (8 × 11 mm i.d.) just before free radical copolymerisation which was initiated by adding N, N, N’, N’-tetramethylethylenediamine (TEMED) (20 *μ*L) and ammonium persulfate (APS) (20 mg). The co-polymerisation was carried out by freezing monomer-initiator mixture at −12 °C for 18 h in cryobath filled with an absolute ethanol. The cryogel columns were thawed at room temperature and washed repeatedly with deionized water to remove unreacted monomers and stored at 4 °C until further use. The resulting cryogel samples were named AAm-AGE cryogels.

To attach affinity ligand protein-A, the epoxy-functionalized monolithic AAm-AGE cryogel columns were first treated with ethylenediamine, then glutaraldehyde (GA). Briefly, 1 mL microporous cryogel columns having 1.5 cm length and 9 mm internal diameter were connected to a peristaltic pump and washed simultaneously with 20 mL of water, at a flow rate of 1 mL/min and then with 0.2 M Na_2_CO_3_ (20 mL). Ethylenediamine solution (0.5 M in 0.2 M Na_2_CO_3_; 30 mL) was recirculated at a flow rate of 1 mL/min for 4 h and washed with water until pH was close to neutral. The cryogel columns were further washed with 20 mL 0.1 M sodium phosphate buffer at pH 7.2 and a solution of glutaraldehyde (5% v/v; 30 mL) in 0.1 M sodium phosphate buffer at pH 7.2 was recirculated through columns using peristaltic pump at a flow rate of 1 mL/min for 5 hours. For coupling protein-A affinity ligand through active aldehyde groups, the solution of protein-A (2 mg/mL; 12 mL) in 0.1 M sodium phosphate buffer at pH 7.2 was recirculated through each column at a flow rate of 1 mL/min at 4 °C for 24 h. Freshly prepared sodium borohydride (NaBH_4_) solution (0.1 in sodium carbonate buffer, 30 mL) at pH 9.2 was recirculated through the cryogel column at a flow rate of 1 mL/min for 3 h to reduce Schiff’s base formed during the coupling reaction.

### Anthrax anti-toxin PANG and Valortim mAb binding and quantification

Anthrax anti-toxin mAbs, PANG (Fraunhofer USA Inc, Delaware, USA) and Valortim (PharmAthene Inc. Maryland, USA) were covalently immobilised on an AAm-AGE cryogel column through protein-A (from Staphylococcus aureus, Sigma, St Louis, MO, USA) as follows. The protein-A surface functionalised AAm-AGE cryogel columns were firstly pre-washed with 50 mM sodium borate solution at pH 8.2. PANG or Valortim antibody solution having a concentration of 2 mg/mL in 50 mM sodium borate was recirculated through cryogel columns using a peristaltic pump at 1.0 mL/min flow rate for 1 h while the pH was maintained at 8.2 throughout the recirculation period. Columns were again washed thoroughly with 50 mM sodium borate followed by 0.2 M triethanolamine at pH 8.2. To form permanent crosslinking between surface protein-A and antibodies, a 5 mL (6.6 mg/mL) solution of crosslinking reagent dimethyl pimelimidate (DMP) in 0.2 M triethanolamine was recirculated through cryogel columns at a flow rate of 1 mL/min for 1 hour at pH 8.2. Columns were washed with the deionised water and the remaining active sites were blocked by washing columns with 5 mL 0.1 M ethanolamine solution at pH 8.2 for 10 minutes. Columns were washed sequentially with deionized water, 1 M NaCl and finally with 0.1 M glycine to remove the un-bound antibody. Finally, columns were washed with deionised water, 1 M NaCl and 0.1 M glycine to remove any antibodies that remain non-covalently bound but not cross-linked, from the columns. Antibody bound cryogel columns were transferred to fridge and stored at 4 °C until further use.

The bicinchoninic acid (BCA) method was used to quantify the amount of protein-A and antibody immobilised on AAm-AGE cryogel columns. Protein-A and antibody bound AAm-AGE columns were dried, finely powdered by grinding and suspended in water by ultra-sonication. Different amounts (20–100 *μ*L) of this suspension were prepared in triplicate and a 2 mL of the BCA solution was added to each tube. This mixture was then incubated at 37 °C for 30 minutes with constant shaking. Unmodified cryogel section samples were used as a control. 3 mL samples were withdrawn with and without centrifuging and transferred to spectrophotometer cuvettes. The absorbance was recorded at 562 nm using a UV spectrophotometer. A standard curve was prepared by plotting the absorbance of samples containing known concentrations of protein and IgG standards from 0, 200, 400, 600, 800, and 1000 *μ*g/mL.

### Porous morphology by SEM

Thin sections of cryogels (1 mm thickness and 9 mm diameter) were frozen at −80 °C for 8 hrs and then transferred to a Christ freeze-dryer to expel water from the cryogel samples for 8 hrs at 0.200 mbar pressure under vacuum. The dried cryogel samples were mounted on an aluminium stub using double sided carbon tape and were sputter-coated with a 4 nm platinum layer for 3 minutes using a Quorum (Q150TES) coater instrument. Scanning electron microscopy (SEM) analysis was carried out at various magnifications (100, 500, and 1000×) using a Zeiss Sigma field emission gun SEM (Zeiss NTS) at an accelerating voltage of 5 kV.

### Pore size distribution by mercury porosimetry and N2-adsorption

The low and high-pressure mercury intrusion measurements were performed to determine macropore (diameter ≤50 nm) size distribution by mechanical intrusion of mercury. Data were anlysed using PoreWin 6.0 software (Quantachrome Instruments, USA). The determination of pore size distribution in the mesoporous range was carried out using an Autosorb-1 gas sorption analyser (Quantachrome Instruments, USA). Prior to the gas adsorption measurement, the freeze dried cryogel samples were out-gassed for 24 hrs at 50 °C to remove any entrapped air or moisture from the pores. The cryogel polymeric structure was preserved by using a relatively low temperature during out-gassing. The adsorbed and desorbed volumes of nitrogen gas by the test samples were measured using an Autosorb-1 gas sorption analyzer instrument (Quantachrome Instruments, USA) at a relative pressure at 77.4 K. Evaluation of sorption data were carried out using Quantachrome data analysis software (Quantachrome ASiQwin). Density Functional Theory (DFT) model was used to determine the mesoporous size distribution.

### PA removal from fresh frozen human plasma and healthy donor whole blood

The structural gene for protective antigen was cloned into the vector pQE30 which has a 6× -Histidine affinity tag, expressed in *E.coli* and purified using a two-step process to achieve a level of purity >95% as described by Stokes *et al*.^45^. The ability of adsorbent monoliths to remove PA toxins was measured using human blood plasma (Cambridge Bioscience, UK) and healthy donor blood (n = 3) spiked with PA. Human blood samples were collected from healthy donors using appropriate informed consent procedures following ethics approval of experimental protocols by the University of Brighton ethics committee (HTA license 12583) and all experiments were performed in accordance with the relevant guidelines and regulations. PA removal studies were carried out using a lab scale experimental flow set up with PA spiked fresh frozen human plasma and freshly withdrawn human whole blood. For each recirculation experiment, syringe like plastic casing was packed with cryogel and connected in line with a peristaltic pump. The inlet and outlet tubing ports were connected to a reservoir containing 20 mL of plasma or blood spiked with anthrax toxin PA. An anthrax toxin adsorption device consisted of a syringe like plastic casing filled with highly interconnected polymeric AAm-AGE cryogel with immobilised Valortim or PANG antibodies capable of capturing anthrax toxin PA by antibody-antigen interaction. AAm-AGE cryogel columns (n = 3) having 9 mm diameter and 1.5 cm length with maximum binding capacities for PANG and Valortim antibodies were chosen to evaluate PA removal from blood *in vitro* and *ex vivo*. A continuously circulating system was used to compare PA removal from plasma and blood by tubing only, unmodified AAm-AGE cryogel, protein-A modified non-specific IgG mAb-bound AAm-AGE cryogel along with PANG and Valortim bound AAm-AGE cryogel columns. A 20 mL reservoir volume of PA spiked plasma and blood at a concentration of 1 mg/mL was circulated through each column pre-wetted with phosphate buffered saline (PBS) at a rate of 2 mL/min. Samples were taken at 5, 10, 30, 45 and 60 minutes timed intervals. Blood samples were centrifuged at 3500 rpm for 15 min and plasma was removed. All samples were stored at −20 °C prior to analysis.

### *In vivo* studies to determine PA removal from rat blood

All animal experiments were performed in accordance with the Animals (scientific Procedures) Act of 1986, which was revised according to the European Directive 2010/63/EU. All animals received humane care according to the criteria outlined in the Guide for the Care and Use of Laboratory Animals (National Institutes of Health publication 86–23; revised 1985). All procedures and protocols were approved by the local University College London (UCL) Animal Welfare and Ethical Review Board (AWERB) and the UK Home Office. Adult male Sprague-Dawley rats (250–300 g, Charles River, UK) were anaesthetised under isofluorane (induction at 5% in oxygen, maintenance at 2% in air for the duration of the study), and had catheters placed in the left carotid artery and right jugular vein. Mean arterial pressure was recorded at hourly intervals (Biopac, US) and core body temperature recorded throughout the study. To maintain body temperature, the animals were placed on regulated heat mats and covered with insulating materials as required. Blood samples for biochemical testing and ELISA were collected into lithium heparin coated tubes for plasma biochemistry prior to, and at hourly intervals throughout the study. PA (List Biological Laboratories, US) was administered by intraperitoneal (IP) injection (100 *μ*g/kg) or infused via the venous catheter for 1 hour at 100 *μ*g/kg. In the filter studies, PA was infused as before for 1 hour prior to connection of the extracorporeal circuit and continued at 40 *μ*g/kg for the remaining 2 hours of the study. After the initial 1-hour infusion period, the arterial catheter was connected to either a Valortim antibody bound or blank control cryogel columns (5 cm by 1 cm (length x diameter)) via a peristaltic pump. The blood was then returned to the animal via the catheter placed into the jugular vein. Flow rates throughout the 2-hour treatment period were set at 2 mL/min with the total circuit volume not exceeding 10% of the circulating blood volume of the animal (ca. 3 mL maximum). The circuit was pre-filled with heparinised saline solution (1000 U/L), with a saline bolus (ca. 3 mL) administered to the animal at the start of treatment to prevent hypotension. At the termination of the study, blood and tissue samples were collected under terminal anaesthesia. Blood samples for biochemistry and PA analysis were stored on ice before centrifugation (3500 rpm, 10 minutes, 4 °C) and stored at −80 °C. Plasma biochemistry (glucose, urea, albumin, total protein, ALT, AST, creatinine) was assessed using an automated COBAS system (Roche, UK) following manufacturer’s instructions.

### An anti-PA sandwich ELISA analysis for PA adsorption

Plasma samples from the adsorption and blood samples from animal model studies underwent a 1 in 2 dilution in assay diluent. Protective antigen concentrations present in the plasma were analysed by using an anti-PA sandwich ELISA. Briefly, a PolySorp Nunc plate (Thermo Sc., Product No 446140) was coated with the PANG antibody at a concentration of 2 *μ*g/mL in 0.1 M bicarbonate-carbonate buffer (0.1 M NaHCO_3_, 0.1 M Na_2_HCO_3_), pH 9.6. 100 *μ*L were added per well and the plate was incubated overnight in the fridge. The plate was washed 6 times 350 *μ*L of PBS + 0.1% Tween-20. The Protective Antigen standard was diluted to a 5–200 ng/mL range in PBS + 0.3 M NaCl + 0.1% Tween-20. 100 *μ*L was added per well and the ELISA plate was incubated for 90 minutes at room temp. The plate was washed 3 times 350 *μ*L of PBS + 0.1% Tween-20. The biotinylated Valortim antibody (prepared using the ImmunoProbe Biotinylation Kit, Sigma, Product No. BK101, according to manufacturer’s instructions) was diluted to a concentration of 4.4 *μ*g/mL in PBS + 0.3 M NaCl + 0.1% Tween-20. 100 *μ*L was added per well and the ELISA plate was incubated at room temperature for 90 minutes. The ELISA plate was washed three times using 350 *μ*L of PBS + 0.1% Tween-20. The Streptavidin-Peroxidase Polymer (Sigma, Product No. S2438) was diluted 1:32000 in PBS + 0.3 M NaCl + 0.1% Tween-20. 100 *μ*L was added per well and incubated the ELISA plate for 90 minutes at room temperature. The plate was washed again three times with 350 *μ*L of PBS + 0.1% Tween-20. 100 *μ*L of TMB (3, 3′, 5, 5′-Tetramethylbenzidine) substrate solution (Thermo Scientific, Product No 34021) was added per well and the plate was incubated in the dark for 15 minutes at room temperature. The reaction was stopped by adding 100 *μ*L of 1 N HCl to each well. The absorbance was read at 450 nm. The absorbance (optical density) was measured using an ELISA plate reader (ELX 800 microplate reader; BioTek Instruments, Inc., Winooski, VT) at 450 nm. All standards and experimental samples were run in triplicate. The standard curve was constructed by plotting absorbance values at 450 nm against the known PA concentrations.

### Statistical analysis

All quantitative data are expressed as mean ± standard deviation. Statistical analysis was performed using GraphPad Prism 5.0 (GraphPad Software, USA). Statistical significance was determined by two-way ANOVA with the Bonferroni post-test. P values less than 0.05 were considered statistically significant.

## References

[1] Dixon TC, Meselson M, Guillemin J, Hanna PC (1999). Anthrax. N Engl J Med.

[2] Petosa C, Collier RJ, Klimpel KR, Leppla SH, Liddington RC (1997). Crystal structure of the anthrax toxin protective antigen. Nat..

[3] Bradley KA, Mourez J, Mourez M, Collier RJ, Young JA (2001). Identification of the cellular receptor for anthrax toxin. Nat..

[4] Rainey GJ, Young JA (2004). Antitoxins: novel strategies to target agents of bioterrorism. Nat Rev Microbiol.

[5] Panchal, R. G. *et al*. Purified bacillus anthracis lethal toxin complex formed *in vitro* and during infection exhibits functional and biological activity. *J Biol Chem* **280**, 10834–9 (2005). 10.1074/jbc.M412210200 15644338

[6] Ezzell, J. J. W. & Abshire, T. G. Serum protease cleavage of bacillus anthracis protective antigen. *J Gen Microbiol* **138**, 543–9 (1992). 10.1099/00221287-138-3-543 1593265

[7] Friedlander AM, Welkos SL, Ivins BE (2002). Anthrax vaccines. Curr Top Microbiol Immunol.

[8] Grabenstein JD (2008). Vaccines: countering anthrax: vaccines and immunoglobulins. Clin Infect Dis.

[9] Inglesby TV (2002). Anthrax as a biological weapon, 2002: updated recommendations for management. Jama.

[10] Jernigan JA (2001). Bioterrorism-related inhalational anthrax: the first 10 cases reported in the united states. Emerg Infect Dis.

[11] Baillie LW (2009). Is new always better than old?: The development of human vaccines for anthrax. Hum Vaccin.

[12] Stepanov AV, Marinin LI, Pomerantsev AP, Staritsin NA (1996). Development of novel vaccines against anthrax in man. J Biotechnol.

[13] Froude, n., J. W., Thullier, P. & Pelat, T. Antibodies against anthrax: mechanisms of action and clinical applications. *Toxins (Basel)* **3**, 1433–52. 10.3390/toxins3111433 PMC3237005 22174979

[14] Little SF, Ivins BE, Fellows PF, Friedlander AM (1997). Passive protection by polyclonal antibodies against bacillus anthracis infection in guinea pigs. Infect Immun.

[15] Hendricks, K. A. *et al*. Centers for disease control and prevention expert panel meetings on prevention and treatment of anthrax in adults. *Emerg Infect Dis* **20** (2014). 10.3201/eid2002.130687 PMC3901462 24447897

[16] Bower WA (2015). Clinical framework and medical countermeasure use during an anthrax mass-casualty incident. MMWR Recomm Rep.

[17] Mett V (2011). A non-glycosylated, plant-produced human monoclonal antibody against anthrax protective antigen protects mice and non-human primates from b. anthracis spore challenge. Hum Vaccin.

[18] Vitale L (2006). Prophylaxis and therapy of inhalational anthrax by a novel monoclonal antibody to protective antigen that mimics vaccine-induced immunity. Infection and Immunity.

[19] Riddle V (2011). Phase i study evaluating the safety and pharmacokinetics of mdx-1303, a fully human monoclonal antibody against bacillus anthracis protective antigen, in healthy volunteers. Clinical and Vaccine Immunology.

[20] Maynard JA (2002). Protection against anthrax toxin by recombinant antibody fragments correlates with antigen affinity. Nat Biotechnol.

[21] Migone TS (2009). Raxibacumab for the treatment of inhalational anthrax. N Engl J Med.

[22] Migone TS (2015). Added benefit of raxibacumab to antibiotic treatment of inhalational anthrax. Antimicrob Agents Chemother.

[23] Rubinson, L. M. P., Corey, A. & Hanfling, D. Estimation of time period for effective human inhalational anthrax treatment including antitoxin therapy. *PLoS Curr* **9** (2017). 10.1371/currents.outbreaks.7896c43f69838f17ce1c2c372e79d55d PMC5555766 28856066

[24] Stearns-Kurosawa DJ, Lupu F, Taylor J, Taylor FB, Kinasewitz G, Kurosawa S (2006). Sepsis and pathophysiology of anthrax in a nonhuman primate model. Am J Pathol.

[25] Tessier J (2007). Contributions of histamine, prostanoids, and neurokinins to edema elicited by edema toxin from bacillus anthracis. Infect Immun.

[26] Iyer JK (2010). Inflammatory cytokine response to bacillus anthracis peptidoglycan requires phagocytosis and lysosomal trafficking. Infect Immun.

[27] Hermanson, G. T. Immobilized affinity ligand techniques (1992).

[28] Spitznagel TM, Clark DS (1993). Surface-density and orientation effects on immobilized antibodies and antibody fragments. Nature Biotechnology.

[29] Rao SV, Anderson KW, Bachas LG (1998). Oriented immobilization of proteins. Mikrochimica Acta.

[30] Gersten DM, Marchalonis JJ (1978). A rapid, novel method for the solid-phase derivatization of igg antibodies for immune-affinity chromatography. J Immunol Methods.

[31] Turkova J (1999). Oriented immobilization of biologically active proteins as a tool for revealing protein interactions and function. J Chromatogr B Biomed Sci Appl.

[32] Lu B, Smyth MR, O'Kennedy R (1996). Oriented immobilization of antibodies and its applications in immunoassays and immunosensors. Analyst.

[33] Babacan S, Pivarnik P, Letcher S, Rand AG (2000). Evaluation of antibody immobilization methods for piezoelectric biosensor application. Biosens Bioelectron.

[34] Anderson GP, Jacoby MA, Ligler FS, King KD (1997). Effectiveness of protein a for antibody immobilization for a fiber optic biosensor. Biosens Bioelectron.

[35] Danczyk R (2003). Comparison of antibody functionality using different immobilization methods. Biotechnology and Bioengineering.

[36] Ingavle GC (2015). Affinity binding of antibodies to supermacroporous cryogel adsorbents with immobilized protein a for removal of anthrax toxin protective antigen. Biomaterials.

[37] Josic D, Buchacher A, Jungbauer A (2001). Monoliths as stationary phases for separation of proteins and polynucleotides and enzymatic conversion. of Chromatography B.

[38] Savina IN, Ingavle GC, Cundy AB, Mikhalovsky SV (2016). A simple method for the production of large 3d macroporous hydrogels for advanced biotechnological, medical and environmental applications. Sci Rep.

[39] Gun’ko VM, Savina IN, Mikhalovsky SV (2013). Cryogels: morphological, structural and adsorption characterisation. Adv Colloid Interface Sci.

[40] Savina IN (2011). Porous structure and water state in cross-linked polymer and protein cryo-hydrogels. Soft Matter.

[41] Zou HF, Huang XD, Ye ML, Luo QZ (2002). Monolithic stationary phases for liquid chromatography and capillary electrochromatography. of Chromatography A.

[42] Zheng, Y. *et al*. Rapid adsorption of proinflammatory cytokines by graphene nanoplatelets and their composites for extracorporeal detoxification. *J of Nanomater*10.1155/2018/6274072.

[43] Kogelmann K, Jarczak D, Scheller M, Druner M (2017). Hemoadsorption by cytosorb in septic patients: a case series. Crit Care.

[44] Buttner S (2014). Extracorporeal virus elimination for the treatment of severe ebola virus disease–first experience with lectin affinity plasmapheresis. Blood Purif.

[45] Stokes MG (2007). Oral administration of a salmonella enterica-based vaccine expressing bacillus anthracis protective antigen confers protection against aerosolized b. anthracis. Infect Immun.

